# Effects of Whey Protein Isolate on Body Composition, Muscle Mass, and Strength of Chronic Heart Failure Patients: A Randomized Clinical Trial

**DOI:** 10.3390/nu15102320

**Published:** 2023-05-16

**Authors:** Elisa M. dos Santos, Annie S. B. Moreira, Grazielle V. B. Huguenin, Eduardo Tibiriça, Andrea De Lorenzo

**Affiliations:** 1 Institute of Heart Edson Saad, Federal University of Rio de Janeiro, Rio de Janeiro 21941-913, RJ, Brazil; 2 Department of Clinical Research, National Institute of Cardiology, Rio de Janeiro 22240-006, RJ, Brazil; 3 Departamento de Nutrição e Dietética, Faculdade de Nutrição Emília de Jesus Ferreiro, Federal Fluminense University, Niterói 24020-140, RJ, Brazil

**Keywords:** heart failure, diet, whey proteins, muscle strength, randomized clinical trial

## Abstract

Heart failure (HF) is associated with a reduction of skeletal muscle mass. Whey protein isolate (WPI) has been beneficial in increasing muscle mass and strength, in addition to improving body composition. The goal of this research was to evaluate the effect of WPI on the body composition, muscle mass, and strength of chronic HF patients. For this purpose, twenty-five patients of both genders with predominantly NYHA I functional class and a median age of 65.5 (60.5–71.0) years were used to conduct a randomized, single-blind, placebo-controlled clinical trial and received 30 g per day of WPI for 12 weeks. Anthropometric measurements, body composition analysis, and biochemical exams were performed at the beginning and end of the study. An increase in skeletal muscle mass was observed in the intervention group after 12 weeks. A reduction in waist circumference, body fat percentage, and an increase in skeletal muscle index was observed when compared to the placebo group. No significant effect on muscle strength was observed after 12 weeks of intervention. These data demonstrate that WPI consumption contributed to the increase of skeletal muscle mass, strength, and reduction of body fat in HF patients.

## 1. Introduction

Heart failure (HF) is one of the main causes of morbidity and mortality worldwide [1]. The impairment of cardiac contractile function leads to several neurohormonal and metabolic disorders, including an imbalance between anabolic and catabolic processes [2], and, in many cases, leads to a reduction of skeletal muscle mass. HF has a significant impact on patient muscle function and body composition, which has been clearly associated with considerable morbidity and institutionalization [3].

Nutritional support in patients with HF should be considered, mainly as a way to prevent progressive weight loss, since restoration of muscle mass may not be achievable [4]. Muscle loss is a strong predictor of frailty and reduced survival in patients with HF, and this process can be mitigated through early nutritional support [5].

Loss of muscle mass affects a large proportion of patients with HF [6], contributing to exercise intolerance and impairment of daily life activities, reduced quality of life, and increased mortality [7,8]. Protein-rich meals stimulate muscle protein synthesis [9], and dietary protein supplementation constitutes a possible intervention for this condition for which no medications are available.

Nutritional recommendations for an older adult (>65 years) population propose an increase in daily protein intake (1–1.2 g/kg/day; 1.2–1.5 g/kg/day in case of inflammatory disease), preferably of high-quality protein (i.e., whey protein), containing large amounts of essential amino acids (EAAs) such as leucine. With advancing age, there is resistance to muscle anabolism, so a daily intake of approximately 25–30 g of high-quality protein is recommended in order to maximize muscle protein synthesis (MPS) [10].

Whey protein isolate (WPI) is a high-quality protein that has been shown to stimulate muscle protein synthesis compared to other protein sources, is highly digestible, and has a high concentration of leucine, which plays an important role in the stimulation of postprandial MPS [11]. Moreover, some studies show that WPI is more effective in stimulating MPS compared to other sources of protein [12,13].

The use of WPI, associated with exercise (especially resistance training), has already been shown to lead to increased muscle protein synthesis and skeletal muscle mass [14], as well as enhanced exercise recovery [15]. Recently, Haß et al. [16], in a pilot study, demonstrated that vibration and resistance exercise plus a high-protein diet based on WPI supplementation (with or without omega-3 fatty acids) increased muscle power in older adults, leading to improved leg strength and chair raise time.

However, many HF patients are unable to exercise for several reasons, ranging from physical restrictions to limitations in the access to cardiac rehabilitation programs, and the effect of the isolated supplementation of WPI in HF patients without concomitant exercise training, although desirable, is still unclear. Therefore, this study aimed to evaluate whether supplementation with WPI, compared to placebo, promotes changes in body composition, especially muscle mass, as well as skeletal muscle strength, in patients with chronic HF.

## 2. Materials and Methods

### 2.1. Patients

This study was approved by the Research Ethics Committee of the National Institute of Cardiology under protocol number 03218512.0.2005.5272. Written informed consent was obtained from all subjects/patients. This was a single-blind, randomized, placebo-controlled clinical trial. Patients with a clinical diagnosis of HF, who had been referred for cardiac rehabilitation by their attending physicians and were awaiting scheduling, were assessed. Inclusion criteria were age ≥50 years, HF class I or II NYHA, clinical stability (symptoms and medications) for at least 4 weeks before inclusion, and left ventricular ejection fraction (LVEF) ≤ 50% (echocardiographically defined). Exclusion criteria were creatinine clearance <50 mL/min/1.73 m^2^, impaired hepatic function (alanine aminotransferase > 150 U/L), hepatic cirrhosis, or allergy to milk proteins.

### 2.2. Supplementation

Participants were randomly allocated to receive supplementation with WPI (30 g/day [27 g protein; 120 kcal/serving]) or placebo, which consisted of maltodextrin (30 g/day [30 g carbohydrates; 120 kcal/ serving]). Patients were instructed to consume the supplements once a day for 12 weeks. The packaging and distribution of the supplements were performed by personnel not involved in the research, and researchers directly involved in patient care and follow-up were unaware of the supplements taken.

### 2.3. Biochemical and Anthropometric Measurements

All patients had fasting blood biochemical evaluation, handgrip-strength assessment, and anthropometric and body composition evaluations. All initial evaluations were repeated after 12 weeks of supplementation.

Systolic blood pressure (SBP) and diastolic blood pressure (DBP) were evaluated using a sphygmomanometer. The SBP and DBP were measured twice by a trained professional, with a 1 min interval between the two measurements, and the average value was used as the patient’s blood pressure.

Blood sampling was performed after a 12 h fasting period. Serum urea, creatinine, glycemia, triglycerides, total cholesterol, and high-density lipoprotein cholesterol were measured by using a biochemistry analyzer (ARCHITECT ci8200, Abbott ARCHITECT**^®^**, Abbott Park, IL, EUA. The LDL-cholesterol (LDL-c) was calculated using the formula by Friedewald et al. [17]. The estimated glomerular filtration rate (eGFR) was calculated by using the equation from the Chronic Kidney Disease Epidemiology Collaboration (CKD-EPI) [18].

The anthropometric evaluation included body weight and height, waist, and hip measurements, as well as body mass index (BMI) calculation. All measurements were made in duplicate and the average values were used. Body composition was evaluated using a bioimpedance system (Inbody 720**^®^**, Bioespace Co. Ltd., Seoul, Republic of Korea), which registered total weight (kg), total skeletal muscle mass (SMM) (kg), total fat mass (kg), and body fat percentage.

Muscle strength was assessed by measuring handgrip strength with a Jamar**^®^** hand dynamometer. Participants were instructed to use the non-dominant hand first, followed by the dominant hand. Three measurements were taken for each hand, with a 10–20 s rest interval between measures to avoid fatigue. The average value of the three measures was used.

Sarcopenia was evaluated by calculating the skeletal muscle index (SMI), considered as the ratio of total SMM in kg to the square of the height in meters [19]. The definition of sarcopenia using the SMI was based on the criteria proposed by Cruz-Jentoft et al., which categorizes sarcopenia into three stages according to gender. Moderate to severe sarcopenia was considered present when SMI was <10.75 kg/m^2^ for men and <6.75 kg/m^2^ for women [20].

### 2.4. Food Consumption

The assessment of the patients’ dietary intake was performed by completing a 24-h dietary recall (R24h). The questionnaire was applied at visits T0 and T12 to longitudinally assess food intake and control possible confounding factors. The domestic measures of the foods consumed described in the 24-h dietary recall were converted to weight or milliliters according to the table of equivalents and home measures. Then these data were entered into the Food Processor version 7.2 program (EshaResearch, Salem, MA, USA, 1998) to calculate the energy intake, macronutrients (carbohydrates, protein, total lipids, and fatty acids), total fiber, and micronutrients.

### 2.5. Statistical Analysis

Randomization occurred in blocks of 2, based on a table of random numbers generated in Openepi**^®^**. The main outcome of the study was the gain of muscle mass. Considering a gain of 500 g in the WP group compared to the placebo, with a standard deviation (SD) of 409.7 g, 11 patients were required in each group (5% alpha, 80% power). The results from a similar study with WP supplementation were used as a reference [21].

Data are presented as the number (percentage) or mean (standard deviation, SD) as appropriate. Categorical variables were presented as values and percentages and compared with a chi-square test. Continuous variables were tested for normality using the Shapiro–Wilk test, were presented as mean and SD or median and interquartile interval, and tested with the Student’s *t*-test or Paired *t*-test (for parametric variables) and Mann–Whitney and Wilcoxon tests (nonparametric variables). Statistical analyses were conducted with IBM**^®^** SPSS**^®^** Statistics software version 23. A value of *p* < 0.05 was considered statistically significant.

## 3. Results

### 3.1. Patient Characteristics and Recruitment

**Figure 1:**
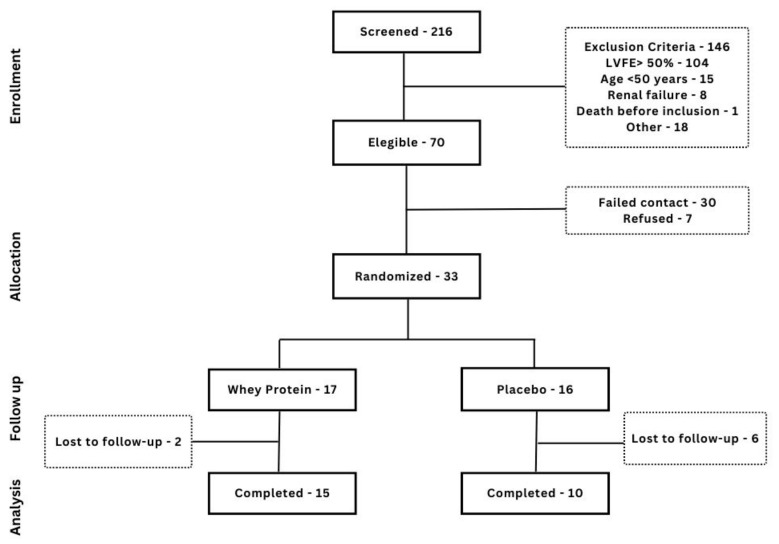
Flowchart of patient inclusion and follow-up.

Thirty-three patients were included, 17 randomized for WPI and 16 for placebo. Eight patients were lost to follow-up, and only 15 and 10 patients, respectively, completed the 12 weeks of the study. The flowchart of patient inclusion and follow-up is depicted in Figure 1. A comparison between patients who completed the 12 weeks of supplementation and those who did not was performed (Supplementary Material), and the only differences found were the prevalence of diabetes (44.0% vs. 87.5%, *p* = 0.032), glycemia (104 (93.5–123.5) vs. 145 (123.0–173.7), *p* = 0.033), and prior myocardial infarction (100% vs. 75%, *p* = 0.01). No significant difference regarding LVEF, NYHA functional class, or BMI was found between patients who completed supplementation and those who did not.

Patients from both groups (WPI or placebo) were most frequently male, with hypertension, dyslipidemia, overweight/obesity, a history of myocardial infarction, mildly reduced mean LVEF, mostly in class I NYHA, and slightly elevated fasting glycemia and triglycerides (Table 1). A high prevalence of sarcopenia (which was moderate to severe in all cases) was found, especially in the placebo group, although no significant difference was found in the prevalence of sarcopenia between groups.

### 3.2. Dietary Assessment

Table 2 depicts the consumption of macro- and micronutrients between the study groups, according to the 24-h recall without considering supplement intake. The analysis of the dietary data showed an adequate percentage of protein intake, but when we evaluated the protein intake per kilogram of body weight per day, it was found to be under appropriate levels for patients with HF [22]. When the supplementation is accounted for in the value of protein intake in T12, it changes to 1.3 ± 0.51 g/kg/day, which is statistically significant compared to the baseline (*p* = 0.007). Carbohydrate intake was close to adequate and both groups had inadequate consumption of lipids [23].

No changes were observed in food consumption for macro- and micronutrients when comparing the baseline and final results of the study, except for sodium consumption, which was reduced in the intervention group throughout the study.

### 3.3. Body Composition

**Figure 2:**
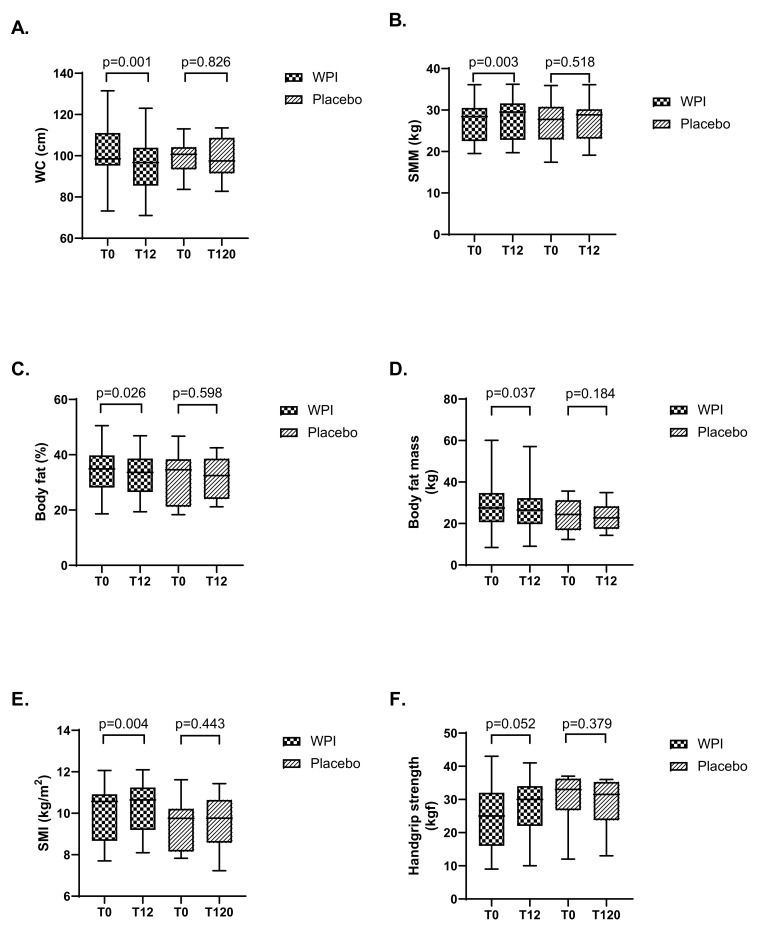
Boxplot of anthropometric and muscle strength data before and after supplementation. (**A**): Waist circumference; (**B**): Skeletal muscle mass; (**C**): Percentual body fat; (**D**): Body fat mass; (**E**): Skeletal muscle index; (**F**): Handgrip strength. Boxplot and 95% CI. Paired *t*-test. SMI, skeletal muscle index; SMM, skeletal muscle mass; WC, waist circumference.

After 12 weeks of WPI supplementation, waist circumference, fat mass, and % fat mass decreased, while skeletal muscle mass and skeletal muscle index increased when compared to placebo (Figure 2). Handgrip strength showed a tendency towards increase (mean 25.7 kgf before and 28.1 kgf after WPI supplementation, *p* = 0.05). Of note, among patients who received the placebo, no significant change was observed. No changes were observed in body mass index for WPI supplementation and placebo when comparing the baseline and final results of the study.

Serum creatinine increased in two groups although it did not show statistical significance. After WPI supplementation, serum urea showed a significant increase when compared to baseline (*p* = 0.045), although this was not clinically relevant (Table 3). The glomerular filtration rate was calculated at baseline and after 12 weeks of supplementation in order to assess the possible negative effect of WPI on renal function of the study participants, but no statistically significant difference was found [79.7 ± 16.6 mL/min/1.73 m^2^ vs. 74.6 ± 15.7 mL/min/1.73 m^2^, showing no statistical difference (*p* = 0.052)].

### 3.4. Other Follow-Up Data

Neither clinical destabilizations (with or without the need for hospital admission) nor changes in patients’ medications were reported during the 12 weeks of the study. No side effects related to gastrointestinal discomfort, such as nausea, vomiting, diarrhea, or constipation, were experienced after drinking the supplement or placebo throughout the study.

## 4. Discussion

In the scenario of HF, loss of muscle mass and strength are of special concern, as they may impair overall quality of life and even survival, and interventions that improve muscle mass and strength are therefore desirable. This study showed that, in patients with chronic HF, the use of WPI, even without concomitant exercise training, was associated with positive outcomes in terms of body composition and skeletal muscle mass. The benefits obtained even in the absence of exercise display an especially important opportunity, as HF patients are often unable to exercise, either due to inherent physical limitations or due to reduced access to cardiac rehabilitation services.

In this study, a high proportion of the study participants had sarcopenia. This is in line with data showing an elevated prevalence of sarcopenic obesity in HF [24,25]. In a recent study, Chen et al. examined the prevalence of sarcopenia in patients with HF and its association with adverse clinical outcomes and found that the overall prevalence of sarcopenia was 31%, and 18% in HF with preserved ejection fraction [26].

Sarcopenia was associated with an increased risk of poor prognosis (mortality and hospitalizations), with a combined hazard ratio of 1.64. Besides these harder outcomes, loss of muscle mass may contribute to frailty and reduction of exercise capacity of patients with HF [27]. Indeed, exercise intolerance is a core component of HF syndrome [28]. Zhang et al., in another large meta-analysis, described a pooled prevalence of sarcopenia in patients with HF of 34%, ranging from 10% to 69% [3]. These numbers underscore the importance of this problem and the need for strategies to increase muscle mass in this patient population.

Regarding dietary composition, the daily protein intake per kilogram of body weight was found to be below appropriate levels for patients with HF at baseline, even though the percentage of protein intake was within normal limits. This reinforces the importance of routine dietary assessment of patients with HF, as nutritional deficits may go unnoticed, although it has long been demonstrated that patients with chronic stable HF often have an inadequate intake of calories and protein [22]. This also underscores the value of protein supplementation for these patients.

Results from a 57-patient nitrogen balance study in non-obese patients with HF, compared with 49 control subjects, suggested that a higher protein goal of 1.1 g/kg or greater may be necessary [22]. Accordingly, the Academy of Nutrition and Dietetics recommends that protein intake should be individualized, but patients with HF should aim for the general population minimum of 0.8 g/kg per day protein intake to prevent cachexia [29].

Interestingly, inadequate consumption of lipids was observed [23]. This may reflect a substitution of fat for carbohydrates, which may be associated with previous concepts concerning the association between fat intake and cardiovascular disease [30], which have been incorporated into medical practice for years and have led to restrictive orientations regarding lipid consumption up to the present time [31].

Some small studies have shown that supplementation of unsaturated fatty acids from dietary sources can have a beneficial effect in patients with HF [32]. A cross-sectional analysis of 23 patients with HF with Preserved Ejection Fraction showed that dietary intake of saturated, monounsaturated, polyunsaturated, and unsaturated fatty acids as a total amount and percentage of daily calories correlated with increased cardiorespiratory fitness as measured by peak VO2 in maximal cardiopulmonary testing [33].

Of note, HF patients were frequently overweight or obese at baseline. It is known that sarcopenia can occur earlier than cachexia in the body-wasting process in HF and may not be associated with weight loss [6]. Muscle damage may be caused by local or systemic factors, such as decreased physical activity, reduced protein and caloric intake, malabsorption, systemic inflammation, oxidative stress, apoptosis, overactivation of the ubiquitin–proteasome system, low muscle blood flow, and endothelial dysfunction [34].

A nutritional solution to minimize the loss of muscle mass in heart failure, even in obese individuals, would be of great relevance in clinical practice. Ideally, it would be interesting for the nutritional supplement not only to stimulate net body protein gain, but also to do so with minimal caloric intake. HF is increasingly being associated with obesity, and a nutritional formulation that significantly increases caloric intake would be undesirable [35].

With WPI, there was a significant increase in skeletal muscle mass (0.6 ± 0.7 kg) after 12 weeks. Prior studies had described the effects of WPI on muscle mass gain, but with associated exercise training [15,36]. The current results are promising as they show results in terms of muscle gain irrespective of the addition of exercise, which may be viewed as a reality in many settings in which cardiac rehabilitation or other methods of supervised training are not available. It is worth noting that WPI did not result in a significant increase in muscle strength, measured by the handgrip. This result is different from a study of elderly patients who participated in high-intensity resistance training, which showed an increase in knee extension strength after supplementation of 24 g WPI [37].

In another report, 23 obese elderly patients with HF with preserved ejection fraction were randomized into three groups: control, whey protein supplementation alone, and whey protein plus light exercise. As in the current study, protein supplementation alone failed to improve physical performance; however, when combined with light exercise, it improved walking speed and quadriceps strength [38]. Differences in functional responses between studies might be attributed either to the lack of exercise training in the current study or to the different muscle groups that were evaluated. Nonetheless, increased muscle mass may be still considered an achieved goal in this scenario, as patients with HF may have benefited from other muscle groups, such as those from the legs, with improved walking capacity, for example, which was not evaluated in this study.

Overall, WPI supplementation has demonstrated beneficial effects on body composition, with a decrease in body fat mass, BMI, and WC, and an increase in lean body mass [39]. In overweight and obese individuals, the benefits of supplementation with WPI on both body composition and cardiometabolic risk by improving the lipid and glycemic profile and reducing body adiposity have been widely demonstrated. A meta-analysis, comprising nine randomized controlled trials and 455 patients, showed the benefit of WPI in reducing body weight, increasing lean body mass, and improving lipid profile and glycemic control [40].

In patients with HF, high-protein diets have also been demonstrated to be advantageous for weight loss and adipose tissue reduction [41]. However, to date, no studies have been found to assess the effect of isolated supplementation with WPI on body composition in this specific patient population. In this study, patients who received WPI had significant reductions in the percentage of body fat, total body fat, and WC. The improvement of these cardiometabolic risk markers may be considered a huge additional gain obtained from WPI, besides the most expected—increased muscle mass. It remains to be explored if these additional favorable effects of WPI supplementation may consistently determine an improvement of outcomes in this particular patient population, who are of high cardiovascular risk.

## 5. Limitations

This study is limited by the small sample size and relatively high dropout rate, especially in the placebo group. However, it offers important new data on the use of WPI for patients with HF, such that that the supplementation of WPI may be useful to increase muscle mass and decrease body fat in a patient population with a high prevalence of overweight/obesity and sarcopenia, who are often unable to exercise.

## 6. Conclusions

In patients with chronic HF with reduced LVEF, 12-week WPI supplementation promoted an improvement of body composition, evidenced by an increase in skeletal muscle mass and a decrease in body fat. Isolated (without accompanying physical exercise) WPI supplementation may be useful for these patients, who are frequently unable to exercise or are limited in their access to supervised exercise programs. The current results may help change the paradigm of the need for exercise during WPI supplementation, which is particularly useful in the specific population of HF patients.

## Figures and Tables

**Table 1:** Baseline characteristics.

	*WPI* (*n* = 17)	Placebo (*n* = 16)	*p*-Value
Age (years)	64 (61–67)	61 (58–78)	0.466
Male	13 (76.5)	13 (81.3)	0.737
Hypertension	13 (76.5)	10 (62.5)	0.383
Diabetes	8 (47.1)	10 (62.5)	0.373
Dyslipidemia	12 (70.6)	9 (56.3)	0.392
Overweight/obesity	15 (88.2)	11 (68.8)	0.171
Prior myocardial infarction	17 (100.0)	14 (87.5)	0.133
Percutaneous coronary intervention	7 (41.2)	5 (31.3)	0.554
Coronary artery bypass grafting	10 (58.8)	5 (31.3)	0.112
NYHA I	15 (88.2)	12 (75.0)	0.325
LVEF (%)	42.0 ± 9.0	44.0 ± 7.5	0.479
Smoking/former smoking	13 (76.5)	14 (87.5)	0.616
BMI (kg/m^2^)	28.6 ± 4.6	26.8 ± 3.5	0.221
WC (cm)	101.7 ± 13.2	100.4 ± 9.2	0.742
Glycemia (mg/dL)	107 (100.0–138.0)	123 (98.0–154.8)	0.605
Triglycerides (mg/dL)	156.4 ± 58.5	176.7 ± 94.2	0.458
Total cholesterol (mg/dL)	159.6 ± 28.3	170.4 ± 41.1	0.382
HDL-cholesterol (mg/dL)	43.9 ± 11.3	40.6 ± 11.8	0.414
LDL-cholesterol (mg/dL)	102 (71.0–125.5)	100 (86.5–130.2)	0.874
Total fat mass (kg)	28.9 (23.9–34.7)	27.6 (18.6–30.9)	0.367
% Body fat	34.9 ± 8.3	32.3 ± 7.8	0.361
SMM (kg)	28.2 ± 5.3	28.6 ± 5.1	0.789
MMI (kg/m^2^)	10.1 ± 1.2	9.8 ± 1.1	0.487
Handgrip strength (kgf)			
Men	31.8 ± 8.0	31.8 ± 6.9	0.979
Women	13.2 ± 3.1	22.3 ± 11.0	0.169
Sarcopenia	7 (41.2%)	10 (62.5%)	0.690
Moderate-severe sarcopenia	7 (41.2%)	10 (62.5%)	0.690

Values are n (%), mean ± SD, or median (25th–75th percentiles). Chi-square test, Student’s *t*-test (BMI, % Body fat, Handgrip strength, HDL cholesterol, LVEF, SMM, Total cholesterol, Triglycerides, WC), Mann–Whitney U test (Age, Glycemia, LDL cholesterol, Total fat mass). BMI: body mass index; HDL: high-density lipoprotein; LDL: low-density lipoprotein; LVEF: left ventricular ejection fraction; MMI: muscle mass index; NYHA: New York Heart Association; SMM: skeletal muscle mass; WC: waist circumference.

**Table 2:** Evaluation of the consumption of macro and micronutrients between the study groups.

	Whey Protein (*n* = 15)			Placebo (*n* = 10)		
	Baseline	12 Weeks	*p*-Value	Baseline	12 Weeks	*p*-Value
Energy intake, kcal/d	1408.4 (840.2–1605.2)	1056.6 (989.6–1565.0)	0.826	1295.0 (965.2–1902.7)	1259.0 (812.2–1673.3)	0.445
Protein, % of energy	18.1 (16.0–22.1)	19.0 (14.8–30.5)	0.363	19.9 (16.8–24.7)	17.6 (14.3–21.5)	0.445
Protein, g.kg BW^–1^. d^–1^	0.8 (0.5–1.0)	0.8 (0.6–1.3)	0.363	0.9 (0.4–1.1)	0.7 (0.5–1.0)	0.203
Carbohydrate, % of energy	60.6 (49.5–65.7)	59.9 (51.3–70.2)	0.397	58.2 (53.2–60.6)	54.3 (49.4–59.7)	0.508
Fat, % of energy	20.4 (14.4–28.5)	18.2 (11.0–22.2)	0.056	23 (16.1–28.4)	26 (21.4–31.5)	0.333
SFA, % of energy	8.3 (4.1–10.8)	5.0 (4.2–8.3)	0.056	8.1 (4.7–12.6)	8.5 (6.7–11.1)	0.799
MUFA, % of energy	5.0 (3.2–7.0)	4.3 (3.2–7.2)	0.683	7.7 (3.2–8.6)	7.5 (5.1–9.3)	0.445
PUFA, % of energy	1.7 (1.4–2.8)	2.0 (1.3–3.3)	0.638	2.3 (1.4–3.7)	2.5 (2.1–3.8)	0.721
n-3 PUFA, g	0.4 (0.2–0.8)	0.3 (0.2–0.8)	0.875	0.6 (0.2–0.7)	0.4 (0.3–0.5)	0.203
n-6 PUFA, g	2.3 (1.1–4.8)	2.4 (1.2–3.3)	0.198	2.4 (1.1–7.0)	3.1 (1.9–4.8)	0.508
Trans fatty acids, g	1.1 (0.2–2.0)	0.4 (0.1–1.6)	0.133	0.4 (0.1–0.5)	0.8 (0.2–1.8)	0.074
Cholesterol, mg	137.6 (71.3–176.0)	157.5 (66.7–242.3)	0.331	123 (64.4–217.2)	164.8 (80.7–207.8)	0.333
Total fiber, g	12.5 (8.8–22.2)	14.6 (11.0–24.2)	0.925	16 (7.1–20.9)	14.6 (8.0–23.6)	0.878
Vitamin A, μg	339.7 (204.0–1191.9)	735.6 (227.0–1867.8)	0.551	696 (301.0–1300.1)	1577.8 (459.6–2443.3)	0.131
Vitamin C, mg	55.4 (9.2–182.4)	54.5 (21.9–134.4)	0.826	32.2 (15.7–67.6)	85.1 (8.1–190.7)	0.333
Vitamin E, mg	1.1 (0.3–2.3)	1.3 (0.8–1.9)	0.778	1.3 (0.7–4.1)	2.4 (1.2–3.5)	0.646
Sodium, mg	1495 (810.5–1979.6)	933 (567.4–1547.4)	0.041 *	1249.9 (686.3–2099)	1159.5 (873.7–1369.1)	0.594
Zinc, mg	4.0 (2.2–7.6)	5.3 (3.4–7.3)	0.124	8.0 (3.8–8.9)	4.1 (2.5–9.9)	0.214

Values are expressed median (25th–75th) percentiles * *p* < 0.05. Wilcoxon test. Considered statistically significant values: *p* < 0.05. MUFA: monounsaturated fatty acid; PUFA: polyunsaturated fatty acids; SFA: saturated fatty acid.

**Table 3:** Biochemical measurements before and after supplementation.

	Whey Protein (*n* = 15)			Placebo (*n* = 10)		
	Baseline	12 Weeks	*p*-Value	Baseline	12 Weeks	*p*-Value
Glucose (mg/dL)	107 (100.0–138.0)	119 (101.0–137.0)	0.570	123 (98.0–154.7)	103.5 (96.5–149.3)	0.674
TC (mg/dL)	157.3 ± 29.3	156.1 ± 41.2	0.826	165.8 ± 36.8	154.7 ± 22.1	0.318
Creatinine (mg/dL)	0.98 ± 0.21	1.06 ± 0.17	0.126	1.08 ± 0.25	1.18 ± 0.53	0.632
Urea (mg/dL)	40 ± 12.8	47.2 ± 22.0	0.045 *	38.6 ± 7.9	43 ± 29.3	0.435
TG (mg/dL)	147.5 ± 44.8	143.3 ± 57.5	0.785	162.2 ± 36.8	218.3 ± 94.0	0.081
HDL-c (mg/dL)	43.2 ± 11.7	44.7 ± 11.5	0.327	41.2 ± 13.8	39.2 ± 12.5	0.244
LDL-c (mg/dL)	96.9 ± 28.4	99.7 ± 37.0	0.597	108.6 ± 27.5	91.5 ± 10.5	0.086

Values are mean ± SD, or median (25th–75th percentiles) * *p* < 0.05. Paired *t*-test; Wilcoxon tests (Glucose). HDL, high-density lipoprotein; LDL, low-density lipoprotein; TC, total cholesterol; TG, triglycerides.

## Data Availability

All datasets generated and analyzed are available from the corresponding author upon reasonable request.

## Supplementary Materials

The following supporting information can be downloaded at: https://www.mdpi.com/article/10.3390/nu15102320/s1, Table S1: Comparison between patients who complete dor not the 12 weeks of supplementation.
