# Supplementary material for: Effects of Whey Protein Isolate on Body Composition, Muscle Mass, and Strength of Chronic Heart Failure Patients: A Randomized Clinical Trial
Source: Nutrients. 2023 May 16;15(10):2320. doi: 10.3390/nu15102320 (PMC10223081; doi:10.3390/nu15102320)
Supplement: Supplementary file 1 [file nutrients-15-02320-s001.zip › nutrients-2236696-supplementary.pdf]

Table S1 – Comparison between patients who complete dor not the 12 weeks of supplementation

	Completed (n=25)	Not completed (n = 8)	p-value
Age (years)	66 (61.0-70.0)	61 (57.2-63.7)	0.053
Male	19 (76.0)	7 (87.5)	0.489
Hypertension	19 (76.0)	4 (50.0)	0.164
Diabetes	11 (44.0)	7 (87.5)	0.032*
Dyslipidemia	18 (72.0)	3 (37.5)	0.077
Overweight/obesity	19 (76.0)	7 (87.5)	0.489
Prior myocardial infarction	25 (100)	6 (75.0)	0.010*
Percutaneous coronary intervention	11 (44.0)	1 (12.5)	0.107
Coronary artery bypass grafting	12 (48.0)	3 (37.5)	0.604
NYHA I	19 (76.0)	8 (100)	0.129
LVEF (%)	42.3±7.4	45.0±10.7	0.426
BMI (Kg/m²)	28.1±4.2	28.2±3.0	0.925
WC (cm)	100.9±12.2	101.6±8.3	0.871
SBP (mmHg)	140.6±22.3	145.7±15.4	0.550
DBP (mmHg)	79.2±19.2	78.5±5.9	0.925
Glycemia (mg/dL)	104.0(93.5-123.5)	145.0(123.0-173.7)	0.033*
Urea (mg/dL)	39.5±11.0	39.5±15.7	0.997
Creatinine (mg/dL)	1.0±0.2	1.0±0.2	0.670
Tryglicerides (mg/dL)	153.4±53.4	206.4±122.7	0.092
Total cholesterol (mg/dL)	160.7±32.0	177.7±42.7	0.237
HDL-cholesterol (mg/dL)	42.4±12.3	41.9±8.7	0.912
LDL-cholesterol (mg/dL)	98(78.0-125.5)	102 (86.5-127.0)	0.911
Quality of life	22(14.0-47.0)	20(12.7-37.0)	0.682

Values are n (%), mean ± SD, or median (25th – 75th percentiles). *: $p<0.05$. Chi-square test, Student t test (BMI, Creatinine, DBP, HDL-cholesterol, LVEF, SBP, Total cholesterol, Tryglicerides, WC, Urea), Mann-Whitney U test (Age, Glycemia, LDL-cholesterol, Quality of life). BMI, body mass index; DBP: Diastolic blood pressure; HDL, high-density lipoprotein; LDL, low-density lipoprotein; LVEF: Left ventricular ejection fraction;NYHA: New York Heart Association; SBP: systolic blood pressure; WC: waist circumference.
